# Supplementary material for: Evaluation of a Dietary Supplementation Combining Protein and a Pomegranate Extract in Older People: A Safety Study
Source: Nutrients. 2022 Dec 6;14(23):5182. doi: 10.3390/nu14235182 (PMC9739468; doi:10.3390/nu14235182)
Supplement: Supplementary file 1 [file nutrients-14-05182-s001.zip › nutrients-2050872-supplementary.pdf]

Supplementary Table 1. Individual compliance (%) in the Control and Test group

Test (n=14)		Control (n=15)	
S1	98.3	S2	95.5
S5	107.8	S3	100.4
S6	107.8	S4	106.1
S7	104.0	S9	109.8
S12	95.0	S10	98.2
S13	104.8	S11	98.7
S14	100.4	S16	94.7
S18	102.0	S17	100.4
S21	94.3	S19	103.1
S22	101.2	S20	90.0
S23	100.1	S24	99.5
S25	100.6	S27	99.3
S26	96.7	S28	112.4
S30	86.4	S29	104.8
		S31	87.0
**Mean**	**100.0**		**100.0**
**SD**	**5.7**		**6.8**
